# Strength–Plasticity Relationship and Intragranular Nanophase Distribution of Hybrid (GNS + SiCnp)/Al Composites Based on Heat Treatment

**DOI:** 10.3390/ma17102460

**Published:** 2024-05-20

**Authors:** Jiajia Zhang, Mingfang Qian, Zhenggang Jia, Xuexi Zhang, Aibin Li, Guisong Wang, Lin Geng

**Affiliations:** School of Materials Science and Engineering, Harbin Institute of Technology, Harbin 150001, China; 22b909046@stu.hit.edu.cn (J.Z.); zgjia@hit.edu.cn (Z.J.); aibinli@hit.edu.cn (A.L.); wangguisong@hit.edu.cn (G.W.); genglin@hit.edu.cn (L.G.)

**Keywords:** aluminum matrix composites, hybrid effect, heat treatment, intragranular nanophase, interface reaction

## Abstract

The distribution of reinforcements and interfacial bonding state with the metal matrix are crucial factors in achieving excellent comprehensive mechanical properties for aluminum (Al) matrix composites. Normally, after heat treatment, graphene nanosheets (GNSs)/Al composites experience a significant loss of strength. Here, better performance of GNS/Al was explored with a hybrid strategy by introducing 0.9 vol.% silicon carbide nanoparticles (SiCnp) into the composite. Pre-ball milling of Al powders and 0.9 vol.% SiCnp gained Al flakes that provided a large dispersion area for 3.0 vol.% GNS during the shift speed ball milling process, leading to uniformly dispersed GNS for both as-sintered and as-extruded (0.9 vol.% SiCnp + 3.0 vol.% GNS)/Al. High-temperature heat treatment at 600 °C for 60 min was performed on the as-extruded composite, giving rise to intragranular distribution of SiCnp due to recrystallization and grain growth of the Al matrix. Meanwhile, nanoscale Al_4_C_3_, which can act as an additional reinforcing nanoparticle, was generated because of an appropriate interfacial reaction between GNS and Al. The intragranular distribution of both nanoparticles improves the Al matrix continuity of composites and plays a key role in ensuring the plasticity of composites. As a result, the work hardening ability of the heat-treated hybrid (0.9 vol.% SiCnp + 3.0 vol.% GNS)/Al composite was well improved, and the tensile elongation increased by 42.7% with little loss of the strength. The present work provides a new strategy in achieving coordination on strength–plasticity of Al matrix composites.

## 1. Introduction

Aluminum (Al), aluminum alloys and composites based on them have been increasingly applied in various fields of technology, e.g., aircraft, space, military, automotive and electronic industries [1,2]. Al and Al alloys have the advantages of good plasticity and low density, but their low strength has become a development limitation. Compared with Al and Al alloys, the elastic modulus and strength of Al matrix composites are obviously improved. In addition, through an appropriate selection of reinforcement phases, the composites always show superior wear resistance compared with that of pure metals [3,4]. Nevertheless, the plasticity of Al matrix composites is seriously lost due to the agglomeration of reinforcement and its poor bonding with the Al matrix. Given this, researchers have used flake powder metallurgy [5,6], severe plastic deformation [7], etc., to promote the reinforcement dispersion, and applied heat treatment [8], chemical vapor deposition [9], etc., to generate appropriate chemical reactions between the reinforcement and the Al matrix, thus improving interfacial bonding. In Al matrix composites, significant breakthroughs in mechanical properties can be achieved only by introducing a small amount of graphene nanosheets (GNS) [10,11,12]. However, GNS/Al composites are faced with several challenges: (1) agglomeration tendency of GNS with a large specific surface area [13,14,15]; (2) difficulty in controlling interface reactions [16,17,18]; and (3) a low work hardening rate during material deformation [8,19,20]. Powder metallurgy is widely used in the preparation of Al matrix composites because of its straightforward process and controllable composition [11,21,22,23]. High-energy ball milling can effectively disperse GNS, but the strong shear effect from hard grinding ball on flexible GNS causes serious structural damage and a significant reduction in composite plasticity [20]. Shift speed ball milling (SSBM) is widely used for GNS dispersion. The key of SSBM is to promote the flattening of Al powder and provide greater dispersion area for GNS [5,24,25,26]. The control of the interface reaction between GNS and the Al matrix is another key factor affecting the properties of composites [27,28]. A proper interface reaction can improve the interface bonding between GNS and Al, which is conducive to a better load transfer role of GNS [21,29,30,31,32,33].

To fully tap the performance potential of Al matrix composites, a novel concept incorporating various reinforcements to achieve enhanced comprehensive properties has been applied [34,35,36]. Generally, the plasticity of Al matrix composites is greatly influenced by the reinforcement distribution. The reinforcement aggregation at grain boundary will induce serious deterioration of grain boundary properties under deformation, leading to early instable fracture of the composites [37,38,39]. The soft lamellar nature of GNS presents challenges in achieving a uniform intragranular distribution while maintaining structural integrity during ball milling, resulting in an insufficient work hardening ability of GNS/Al composites [8]. Studies have shown that [40,41,42], composites with nano hard particles inside Al grains can achieve high tensile strength while maintaining high plasticity, and nano particles can also introduce high-density dislocations inside the grains, significantly improving the work hardening rate of the composites. Therefore, the introduction of a nano-scale secondary phase into GNS/Al composites by a hybrid reinforcement approach is expected to realize the comprehensive performance breakthrough of GNS/Al composites.

In this study, 0.9 vol.% silicon carbide nanoparticles (SiCnp) were introduced into 3.0 vol.% GNS/Al composites. Al flakes with a large specific surface area, favorable for GNS dispersion, were obtained by pre-ball milling of SiCnp and Al powders. Simultaneous enhancement of strength and plasticity of as-extruded (0.9 vol.% SiCnp + 3.0 vol.% GNS)/Al composite was achieved after heat treatment. It is noteworthy that the intragranular distribution of SiCnp was obtained due to recrystallization and grain growth after high-temperature heat treatment of the as-extruded composite at 600 °C for 60 min and nanoscale Al_4_C_3_ was formed between GNS and Al. Compared to 3.0 vol.% GNS/Al composite with significantly reduced strength, (0.9 vol.% SiCnp + 3.0 vol.% GNS)/Al exhibited stable strength with improved plasticity and work hardening ability. Different from the existing status of strength loss after heat treatment in the previous GNS/Al composites, the positive influence of the intragranular distribution of nanoparticles and appropriate interfacial reactions during high-temperature heat treatment process leads (SiCnp + GNS) /Al to show superior strength stability. This study presents a novel approach to enhance the GNS dispersion and achieve a performance breakthrough in GNS/Al composites.

## 2. Experimental Methods

**Figure 1:**
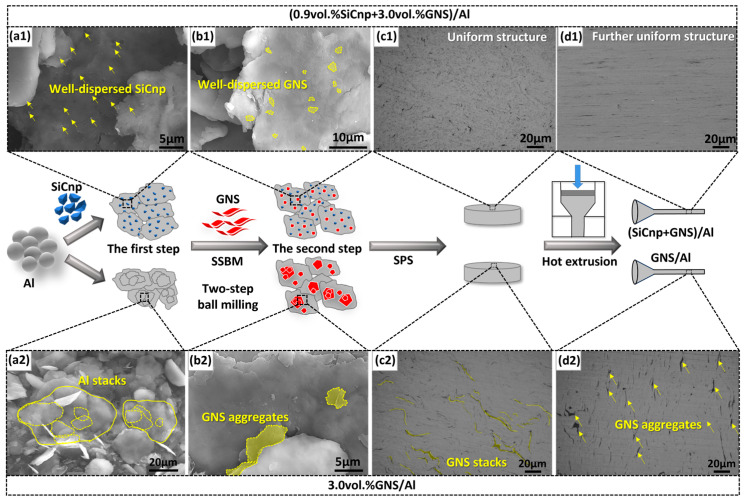
(0.9 SiCnp + 3.0 GNS)/Al and 3.0 GNS/Al composites’ preparation processes and corresponding microstructure characteristics at each stage. (**a1**,**b1**,**c1**,**d1**) (0.9 SiCnp + 3.0 GNS)/Al with uniformly distributed reinforcements; (**a2**,**b2**,**c2**,**d2**) 3.0 GNS/Al with obvious aggregates of GNS. (**a1**,**a2**) morphologies of Al powders after the first step ball milling; (**b1**,**b2**) morphologies of composite powders after the second step ball milling; (**c1**,**c2**) metallographic morphologies of the as-sintered composites; (**d1**,**d2**) optical morphologies of as-extruded composites.

Detailed experimental details for the preparation of a (SiCnp + GNS)/Al composite have been published in previous studies [43] and are briefly described here (Figure 1). A two-step ball milling process was employed: (1) Al powders (average particle size~10 μm) and 0.9 vol.% SiCnp (average particle size~60 nm) were pre-milled at 200 rpm for 10 h to obtain Al flakes with large specific surface area on which SiCnp was uniformly dispersed; (2) set 3.0 vol.% GNS was added into Al-SiCnp powders for SSBM, and finally (SiCnp + GNS)/Al composite powder with uniform reinforcement distribution was obtained. The composite powder was sintered by SPS and the (0.9 vol.% SiCnp + 3.0 vol.% GNS)/Al composite was obtained by hot extrusion, which is abbreviated as (0.9 SiCnp + 3.0 GNS)/Al in the following contents. The full names and abbreviations of the composites are summarized in Table 1. The preparation process of the control group 3.0 GNS/Al composite was the same; the only difference was that in the first ball milling step, only pure Al powder was milled. The two groups of composites were heat-treated at 600 °C for 30 min and 60 min to control the strength and plasticity.

The relative densities of the composites, as determined by the Archimedes method, all exceeded 99%. Scanning electron microscopy (SEM, Merlin Compact) was used to observe the morphologies of milled powders and microstructures of the composites. Optical microscopy (OM, SZ61TR, Olympus-IMS, Houston, TX, USA) was used to observe the distribution of GNS in composites. Electron backscatter diffraction (EBSD, Zeiss Gemini560, Jena, Germany) was used to determine the grain morphologies, where the EBSD orientation data were acquired with a step size of 0.15 μm. The specimens for EBSD analysis were prepared by cross section ion polishing on an ion grinding instrument (IM4000 II, Hitachi High-Tech, Tokyo, Japan). Post-processing of EBSD data was performed using channel 5 commercial software (Oxford Instrument, HKL A/S 2007, Abbington, UK). The distribution of geometrically necessary dislocations (GNDs) and the density of geometrically necessary dislocations ($\rho_{GND}$) were obtained by a kernel average misorientation (KAM) distribution diagram. Transmission electron microscopy (TEM, Talos F200x, Thermo Fisher Scientific, Waltham, MA, USA) was used to observe the microstructures, especially the distribution of the nanophases, and the lattice strain distribution of the Al matrix was obtained through geometric phase analysis (GPA). The mechanical properties of dog-bone-shaped composite samples were tested at room temperature using AG-Xplus 20 KN electronic universal testing machine (tensile rate 0.5 mm/min, ANALIT Ltd., Saint Petersburg, Russia).

## 3. Results and Discussion

Figure 1 shows the comparison of microstructure characteristics between (0.9 SiCnp + 3.0 GNS)/Al and 3.0 GNS/Al composites at the stages of ball milling, sintering and extrusion. Previous studies have shown that the addition of SiCnp can effectively promote the uniform dispersion of high-content GNS [43]. During the first ball milling step, SiCnp significantly facilitated the flattening of the spherical Al powders (Figure 1(a1)), avoiding the stacking of Al powders without SiCnp (Figure 1(a2)). It can also be observed from the composite powders after two-step ball milling that GNSs were uniformly dispersed on the surface of Al flakes with SiCnp (Figure 1(b1)), whereas significant GNS aggregates were observed in the control group (Figure 1(b2)). Effective GNS dispersion was achieved for both as-sintered and as-extruded (0.9 SiCnp + 3.0 GNS)/Al, as depicted in Figure 1(c1,c2,d1,d2), in comparison to 3.0 GNS/Al. In summary, the addition of SiCnp effectively and successfully controlled the morphology of the Al powder, where uniform GNS dispersion was realized.

**Figure 2:**
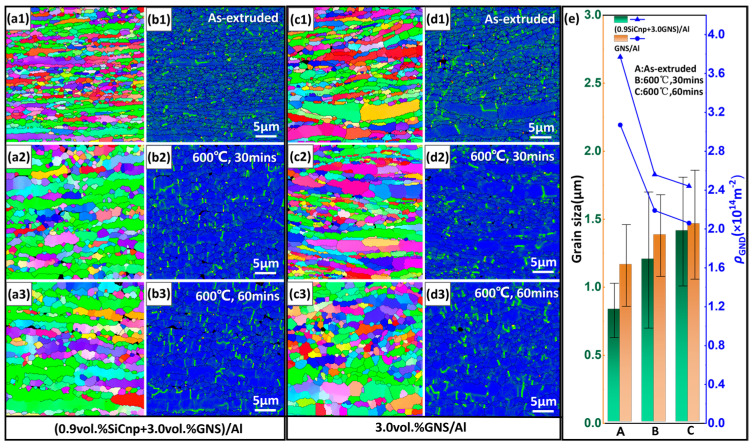
Microstructure characteristics of (0.9 SiCnp + 3.0 GNS)/Al and 3.0 GNS/Al composites before and after heat treatments. (**a1**–**a3**,**b1**–**b3**) Grain morphologies and GND distribution of (0.9 SiCnp + 3.0 GNS)/Al; (**c1**–**c3**,**d1**–**d3**) grain morphologies and GND distribution of 3.0 GNS/Al; (**e**) statistics of grain size and $\rho_{GND}$ variations in composites. Numbers 1, 2 and 3 correspond to the composites after extrusion and heat treatment at 600 °C for 30 min and 60 min, respectively.

Heat treatments were performed on both (0.9 SiCnp + 3.0 GNS)/Al and 3.0 GNS/Al composites at 600 °C for 30 and 60 min. The microstructure of the composites in different states is shown in Figure 2. The specific values of Al grain size and $\rho_{GND}$ were summarized and are listed in Table 2. As shown in Figure 2(a1,a2,a3,c1,c2,c3), SiCnp had a significant impact on grain refinement. Owing to the uniform dispersion of GNS, (0.9 SiCnp + 3.0 GNS)/Al possessed a more uniform grain size distribution than that of 3.0 GNS/Al. After 30 min of heat treatment, (0.9 SiCnp + 3.0 GNS)/Al (Figure 2(a2)) showed more obvious recrystallization than 3.0 GNS/Al (Figure 2(c2)), and the grain growth rate of the former was significantly higher (Figure 2e). During the heat treatment process, the addition of reinforced particles may accelerate recrystallization due to the particle-stimulated nucleation or retard recrystallization through its pinning of the grain boundaries [40,44]. Previous studies [45] have shown that when *V/Dp* > 0.2 µm^−1^, the effect of particles changes from accelerating recrystallization to delaying it, where *V* is the particle volume fraction and *Dp* is the particle diameter. In the present work, the calculated *V/Dp* is 0.15 µm^−1^ (*V*: 0.9 vol.%, *Dp*: 60 nm), leading to accelerated recrystallization of Al grains during heat treatment. In addition, as shown in Table 2, after heat treatment for 30 min, the fitting ellipse aspect ratio of (0.9 SiCnp + 3.0 GNS)/Al decreased significantly from an average value of 3.9 to 2.4, while that of 3.0 GNS/Al, however, only decreased from 3.5 to 3.29. This also explains the increase in the proportion of recrystallized grains in (0.9 SiCnp + 3.0 GNS)/Al. The addition of SiCnp provided more nucleation sites for Al grains, promoted the recrystallization of Al grains during heat treatment, and increased the possibility of Al grains being wrapped by grain boundaries during recrystallization.

The long-range stress (back stress) exerted by GNDs plays an important role in strain hardening, strengthening and thus improving mechanical properties [46,47,48,49]. According to Figure 2(b1,b2,b3,d1,d2,d3), the addition of SiCnp led to a significantly higher $\rho_{GND}$ in (0.9 SiCnp + 3.0 GNS)/Al compared to 3.0 GNS/Al, which enhanced the material deformation resistance during loading and contributed to the material strengthening. As shown in Figure 2e, after the heat treatments, the $\rho_{GND}$ in composites decreased significantly, which was due to the activation of dislocation movement caused by grain growth and recrystallization. Therefore, appropriate heat treatment reduced the dislocation density in the Al matrix, and improved the continuity of the Al matrix to improve the plasticity of the composites.

**Figure 3:**
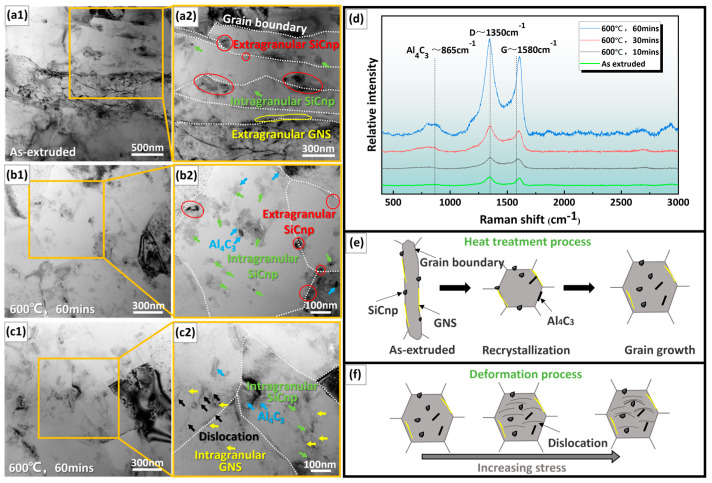
Reinforcement distribution and Al_4_C_3_ generation within (0.9 SiCnp + 3.0 GNS)/Al composites in as-extruded and heat-treated states. (**a1**,**a2**) TEM bright field images of as-extruded sample; (**b1**,**b2,c1**,**c2**) TEM bright field images of sample after heat treatment at 600 °C for 60 min; (**d**) Raman spectra reflecting the generation of Al_4_C_3_ under different states; (**e**) schematic diagram of microstructure evolution during heat treatment; (**f**) schematic diagram of dislocation movement upon loading of the heat-treated (0.9 SiCnp + 3.0 GNS)/Al composite.

The intragranular distribution of reinforcements can improve the continuity of the Al matrix and reduce the occurrence of grain boundary brittleness caused by the aggregation of reinforcements at grain boundaries [39]. As shown in Figure 3(a1,a2), GNS and SiCnp in as-extruded (0.9 SiCnp + 3.0 GNS)/Al were primarily distributed at the grain boundaries elongated along the extrusion direction, with some SiCnp distributed inside the Al grains. As for hard particles, SiCnp is easier to be encapsulated by grain boundaries compared to flexible GNS. After heat treatment at 600 °C for 60 min (Figure 3(b1,b2,c1,c2)), the Al grains significantly grew and transformed into equiaxed grains, and the amounts of intragranular SiCnp and GNS in the Al matrix increased significantly (Figure 3(b2)). Furthermore, Al_4_C_3_ was found both at the grain boundaries and in the grains. With the extension of heat treatment time, the characteristic peak intensity of Al_4_C_3_ significantly increased (Figure 3d). During the heat treatment process (Figure 3e), the Al grains were recrystallized, surrounding the nano phase to its interior, and improving the continuity of the Al matrix. As shown in the schematic diagram in Figure 3f, during the deformation process, the nanophase prevented the dislocation slip, resulting in dislocation accumulation, and increased the deformation resistance of the composite. The overall effect is to improve the mechanical properties.

**Figure 4:**
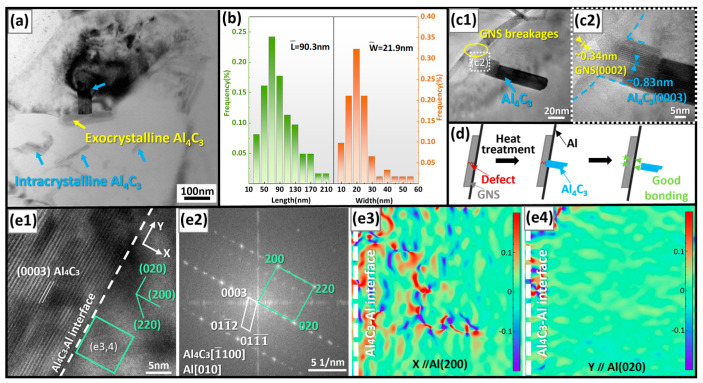
Distribution of Al_4_C_3_ in (0.9 SiCnp + 3.0 GNS)/Al after heat treatment at 600 °C for 60 min. (**a**) TEM bright field image showing distribution of Al_4_C_3_; (**b**) statistics on the length and width of Al_4_C_3_; (**c1**,**c2**) TEM bright field images showing Al_4_C_3_ growing at GNS defect; (**d**) schematic diagram of Al_4_C_3_ growth according to (**c1**,**c2**); (**e1**,**e2**) HRTEM images and (**e3**,**e4**) GPA analysis of Al_4_C_3_/Al interface.

The distribution of Al_4_C_3_ in (0.9 SiCnp + 3.0 GNS) /Al after heat treatment at 600 °C for 60 min was determined. As shown in Figure 4a, Al_4_C_3_ existed both at the grain boundaries and within the grains. Size statistics on Al_4_C_3_ observed in multiple regions were performed. The average length and width values of Al_4_C_3_ were 90.3 and 21.9 nm, respectively (Figure 4b). Research has shown that micrometer-scale Al_4_C_3_ significantly deteriorates the mechanical properties of composites, but nanoscale Al_4_C_3_ is beneficial for increasing the interface shear stress and improving the strength of the composites [50]. It can be seen from Figure 4(c1,c2) that Al_4_C_3_ grew from GNS breakages at a certain angle. Schematically, as shown in Figure 4d, Al_4_C_3_ tended to generate at the GNS defect site, resulting in a certain degree of chemical bonding between GNS and Al, thus improving the bonding strength.

HRTEM images (Figure 4(e1,e2)) of the Al_4_C_3_–Al interface were used for GPA analysis (Figure 4(e3,e4)). As shown in Figure 4(e1,e2), it can be observed that (0003)Al_4_C_3_ is parallel to (020)Al, and the mismatch between the (0003)Al_4_C_3_ and (020)Al was calculated as follows [51]: $\delta =\frac{\left|d_{\left(0003\right)Al4C3}-{4d}_{\left(020\right)Al}\right|}{{4d}_{\left(020\right)Al}}=\frac{\left|0.83-4*0.20\right|}{0.20}=3.7\%$. According to Bramfitt’s lattice matching theory [52], the calculations resulted in a coherent interface between the (0003)Al_4_C_3_ and (020)Al. As shown in Figure 4(e3,e4), the lattice strain distribution of the Al matrix was the largest in the <200>Al crystal direction, being perpendicular to the interface of Al_4_C_3_–Al. Furthermore, the degree of lattice distortion increased as the region approached Al_4_C_3_. The large lattice strain at the Al_4_C_3_–Al interface also indicated a good combination between them [53]. Through proper heat treatment, the defects of GNS can be effectively consumed. In addition, intragranular Al_4_C_3_ was also utilized as nano reinforcement to improve the dislocation density in composites while ensuring the continuity of the Al matrix. This contributed to a synchronous improvement in the strength and plasticity of the present hybrid composites.

**Figure 5:**
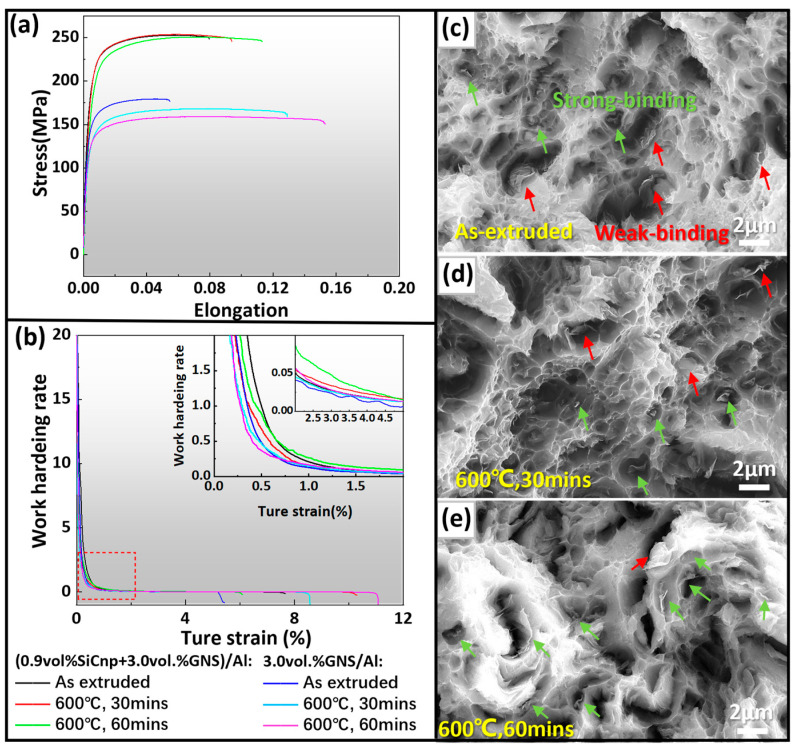
Mechanical properties and fracture morphologies of (0.9 SiCnp + 3.0 GNS)/Al and 3.0 GNS/Al composites at different states. (**a**) Tensile mechanical property curves; (**b**) work hardening rate curves; (**c**–**e**) fracture morphologies of (0.9 SiCnp + 3.0 GNS)/Al after extrusion, and heat treatment at 600 °C for 30 min and 60 min, respectively.

The mechanical property curves of (0.9 SiCnp + 3.0 GNS)/Al and 3.0 GNS/Al composites at different states are shown in Figure 5a,b, and the specific data are summarized in Table 3. In previous studies, heat-treated GNS/Al composites usually show a significant decline in strength [8,50,54,55]. For instance, Li et.al. [8] found that the strength of a 2 wt.% GNS/Al composite significantly reduced by 27% after heat treatment at 600 °C. By comparison, the addition of SiCnp significantly improved the strength of the composites in the present work. As shown in Figure 5a, as expected, after high-temperature heat treatment at 600 °C, the strength of 3.0 GNS/Al decreased significantly compared with that of the as-extruded state. On the other hand, the strength of the heat-treated (0.9 SiCnp + 3.0 GNS)/Al remained highly stable. When the heat treatment time was extended to 60 min, the elongation of (0.9 SiCnp + 3.0 GNS)/Al increased to 11.3%, which was 42.7% higher than that of the as-extruded state, while the strength remained almost unchanged. As shown in Figure 5b, the work hardening rate values of (0.9 SiCnp + 3.0 GNS)/Al at different states were significantly higher than those of 3.0 GNS/Al. At the initial deformation stage (true strain = 0~0.5%), the work hardening rate values of heat-treated (0.9 SiCnp + 3.0 GNS)/Al and 3.0 GNS/Al were both lower than those of the as-extruded ones. This was mainly due to the growth in grain size and the significant reduction in dislocation density in the matrix after heat treatment. With true strain increased to 0.6%, the work hardening rate value of 60 min heat-treated (0.9 SiCnp + 3.0 GNS)/Al became higher than that of the as-extruded state, and the same phenomenon occurred in the 30 min heat-treated one when the true strain reached 2%, as shown in Figure 5b.

(0.9 SiCnp + 3.0 GNS)/Al exhibits a simultaneous increase in strength and plasticity and excellent performance stability after high-temperature heat treatment over 3.0 GNS/Al composites. The reasons are multifold. Firstly, the SiCnp addition resulted in significant grain refinement, which led to fine grain strengthening (Figure 2). On the other hand, proper heat treatment promoted the interface reaction between GNS and Al, leading to better chemical bonding, thus enhancing the load transfer strengthening effect. Moreover, the nanoscale Al_4_C_3_ generated can also play a strengthening role as reinforcing nanoparticles (Figure 4b). Most importantly, due to the recrystallization and grain growth of the Al matrix during heat treatment, intragranular distribution of SiCnp and of Al_4_C_3_ nanoparticles was attained (Figure 3(b1,b2,c1,c2)). Intragranular nanophase distribution introduces higher dislocation density without compromising matrix continuity in the process of deformation, thereby preserving the material’s plasticity. Given this, heat-treated (0.9 SiCnp + 3.0 GNS)/Al exhibited significantly improved plasticity and work hardening ability while maintaining material strength stability, as displayed in Figure 5a,b. In addition, conventional hybrid-reinforced Al matrix composites are usually fabricated by direct ball milling of the two reinforcements with Al powder, which increases the difficulty of uniform dispersion of the reinforcements, leading to a significant increase in strength but with a loss of plasticity [56,57,58,59]. For instance, Ghazaly et al. [56] prepared a (GNS + SiC)/Al composite by ball milling with simultaneous addition of GNS and SiC to Al powder. The hardness and tensile strength of the obtained composite increased by 162% and 20.69%, respectively, but the plasticity decreased significantly from 9.8% to 3.4%.

Figure 5c–e are fracture surface morphologies of (0.9 SiCnp + 3.0 GNS)/Al at different states, where a large number of dimples with GNS distributed inside them on the fracture surface can be observed for all states. However, as shown in Figure 5c of the as-extruded state, in the majority of the dimples, the GNS and dimple wall were in separation and pull-out states, indicating weak interfacial bonding, thus resulting in lower elongation of the composite. When the annealing time is extended to 30 min, as shown in Figure 5d, the debonding phenomenon is greatly reduced, and only a small amount of GNS is pulled out, indicating that the interface between GNS and the Al matrix is strengthened. When the annealing temperature is extended to 60 min (Figure 5e), the vast majority of GNSs are located in the fracture dimples and present a fracture state. After proper heat treatment, GNS and Al produce trace interfacial reaction, and the interfacial bond of GNS-Al is transformed from a mechanical bond to a mechanical and chemical bond with higher degree. The good combination of GNS with the Al matrix helped to give full play to its strengthening effect and can effectively transfer the load during the stretching process, leading to a simultaneous increase in strength and plasticity of the (0.9 SiCnp + 3.0 GNS)/Al composite.

## 4. Conclusions

This work explored the comprehensive mechanical properties of GNS/Al with a hybrid strategy by introducing SiCnp into the composite and performing proper heat treatment to optimize the reinforcement distribution and adjust the interface reaction. The main outcomes are as follows:

(1) Pre-ball milling with SiCnp avoided the stacking of Al powders and gained Al flakes that provided a large dispersion area for GNS, leading to effective GNS dispersion for both as-sintered and as-extruded (0.9 SiCnp + 3.0 GNS)/Al.

(2) Obvious grain refinement was obtained in (0.9 SiCnp + 3.0 GNS)/Al. The incorporation of SiCnp promotes the grain refinement and the recrystallization of aluminum grains during heat treatment, and intragranular SiCnp distribution was realized through high-temperature heat treatment at 600 °C for 60 min.

(3) Proper heat treatment can promote stronger bonding of GNS with the Al matrix, and effectively inhibit debonding between them during the tensile process. After high-temperature heat treatment at 600 °C for 60 min, nanoscale Al_4_C_3_ was obtained both at grain boundaries and inside the grains, with a 3.7% mismatch between (0003) Al_4_C_3_ and (020)Al.

(4) In comparison to the GNS/Al composite, the elongation of the hybrid (GNS + SiCnp)/Al increased by 42.7% while maintaining stable strength and improved work hardening capability.

## Figures and Tables

**Table 1:** Full names and abbreviations of the composites.

Full Name	Abbreviation
(0.9 vol.% SiCnp + 3.0 vol.% GNS)/Al	(0.9 SiCnp + 3.0 GNS)/Al
3.0 vol.% GNS/Al	3.0 GNS/Al

**Table 2:** Statistics of average grain size, fitted ellipse aspect ratio and $\rho_{GND}$ of (0.9 SiCnp + 3.0 GNS)/Al and 3.0 GNS/Al composites at different states.

Specimen	(0.9 SiCnp + 3.0 GNS)/Al			3.0 GNS/Al		
	Average Grain Size (μm)	Fitted Ellipse Aspect Ratio	$\rho_{GND}($/m^2^)	Average Grain Size (μm)	Fitted Ellipse Aspect Ratio	$\rho_{GND}($/m^2^)
As-extruded	0.8 ± 0.2	3.9 ± 2	3.8 × 10^14^	1.2 ± 0.3	3.5 ± 1.8	3.1 × 10^14^
600 °C, 30 min	1.2 ± 0.5	2.4 ± 1.5	2.6 × 10^14^	1.5 ± 0.3	3.29 ± 1.5	2.2 × 10^14^
600 °C, 60 min	1.4 ± 0.4	2.24 ± 1.3	2.4 × 10^14^	1.5 ± 0.4	2.3 ± 1.2	2.1 × 10^14^

**Table 3:** Yield strength (YS), ultimate tensile strength (UTS), elongation (δ) of (0.9 SiCnp + 3.0 GNS)/Al and 3.0 GNS/Al at different states.

Specimens	States	YS (MPa)	UTS (MPa)	δ (%)
(0.9 SiCnp + 3.0 GNS)/Al	As-extruded	207.2 ± 2.3	252.9 ± 5.4	7.9 ± 0.5
	600 °C, 30 min	201.9 ± 3.6	253.9 ± 3.5	9.4 ± 0.7
	600 °C, 60 min	190.7 ± 4.1	250.7 ± 6.7	11.3 ± 1.0
3.0 GNS/Al	As-extruded	138.6 ± 8.1	179.4 ± 1.5	5.4 ± 0.3
	600 °C, 30 min	125.1 ± 4.9	168.2 ± 7.6	12.7 ± 2.1
	600 °C, 60 min	122.3 ± 6.8	159.1 ± 5.8	15.0 ± 2.8

## Data Availability

The data related to this work can be obtained from the corresponding author upon reasonable request.

## Abbreviations

Full name	Abbreviation
Aluminum	Al
Graphene nanosheets	GNS
Silicon carbide nanoparticles	SiCnp
Shift speed ball milling	SSBM
Geometrically necessary dislocations	GNDs
Density of geometrically necessary dislocations	$\rho_{GND}$
Kernel average misorientation	KAM
Geometric phase analysis	GPA
